# Measuring the Facial Plate of Bone in the Upper Anterior Teeth Utilizing Cone Beam Computed Tomography at King Abdulaziz University, Jeddah, Saudi Arabia

**DOI:** 10.7759/cureus.29453

**Published:** 2022-09-22

**Authors:** Badr Othman, Talal Zahid, Hanadi Khalifa, Abdulmajeed Afandi, Nawaf A Alshehri, Ahmed Sait, Sultan Abdoun

**Affiliations:** 1 Periodontology, Faculty of Dentistry, King Abdulaziz University, Jeddah, SAU; 2 Radiology, Faculty of Dentistry, King Abdulaziz University, Jeddah, SAU; 3 General Dentistry, Faculty of Dentistry, King Abdulaziz University, Jeddah, SAU

**Keywords:** esthetics zone, maxillary anterior teeth, dental implants, cone beam ct scan, radiology, dentistry

## Abstract

Background and aim

Radiographic assessment is an important diagnostic tool in dental practice.Cone beam computed tomography (CBCT) is among the most important imaging examinations. By providing multiplanar visualization of the maxillofacial region, CBCT enables practitioners to assess various conditions three-dimensionally. CBCT is utilized in different fields within dentistry, including oral and maxillofacial surgery, endodontics, orthodontics, periodontics, implant dentistry, and others. Having access to accurate 3D images is crucial in implant dentistry. This study aimed to measure the crestal bone height loss and facial alveolar bone thickness in the maxillary anterior teeth using CBCT to investigate its effect on surgical planning for dental implant placement in adult patients.

Material and methods

CBCT scans (N = 119) of adults, aged 18-65 years, with bilateral permanent maxillary anterior teeth present were included in this retrospective study. The mean alveolar bone plate thickness and crest bone height loss adjacent to the maxillary anterior teeth were measured and differences were examined.

Results

The results suggest that additional care and assessment of dental implant placement should be considered when replacing the permanent lateral incisors and canines. The frequency of fenestrations and dehiscence is higher in older adults. Possible management includes guided bone regeneration or “pink restorative solutions.”

Conclusion

CBCT analysis to assess the bone morphology surrounding “hopeless” maxillary anterior teeth is important to ensure proper diagnosis and management, including the use of dental implants.

## Introduction

Radiographic examination is an important diagnostic step in dental practice. Cone beam computed tomography (CBCT) is one of the most useful imaging examinations. By providing multiplanar visualization of the maxillofacial region, CBCT enables practitioners to assess various conditions in detail. CBCT provides multiple advantages for assessing the hard tissues of the craniofacial region [1].

CBCT images are of high quality and accuracy. CBCT also requires less radiation exposure than multidetector CT (MDCT) imaging, by approximately ten times. CBCT is utilized in different disciplines in dentistry, including oral and maxillofacial surgery, endodontics, orthodontics, periodontics, implant dentistry, and others. Having access to accurate 3D images is vital in implant dentistry. CBCT assessment provides reliable information that facilitates proper case selection and more accurate evaluation of the bone quality and quantity and provides a guide for correct implant placement, which, in turn, will reduce the risk of implant failure [2].

Implant dentistry has gained the attention of many practitioners and is commonly used for anterior teeth replacement. The management of anterior teeth loss is challenging due to the possibility of soft tissue recession and bone resorption in the esthetic area. Careful assessment of the bone anatomy and soft tissue type and contour is mandatory when planning implant placement. CBCT is recommended for such an assessment. The individual factors and conditions of each patient must be considered and assessed. These factors include the facial type. As reported by Gracco et al., an individual with a short face tends to have greater alveolar bone thickness compared with those who have a long facial type. Other factors, such as the jaw protrusion degree and the inclination of incisors, also contribute to alveolar bone thickness [3].

CBCT is especially useful as a prognostic tool for determining the alveolar bone thickness, and identifying the possibility of any pre or post-surgical complication upon implant placement [4]. Previous studies have reported on various issues in relation to the esthetic outcome and possible complications and limitations [5]. For example, a patient with a thin biotype represents a reduced facial plate thickness when compared to a patient with a thick biotype. The use of dental implants as a conservative treatment for replacing missing anterior teeth has become very popular due to their better functional esthetic outcome when compared to management using fixed or removable prostheses [6].

Vertical and horizontal bone loss is expected after teeth extraction [7]. Management of patients with “hopeless” or missing anterior teeth via dental implants may vary depending on the alveolar bone thickness and the reduction in crestal bone height. Preservation of the existing bone is always preferable to guided bone regeneration following teeth extraction. Immediate dental implants with immediate provisional restoration can preserve and minimize the loss of soft and hard tissues [8].

Faculty of Dentistry, King Abdulaziz University is one of the major academic and clinical institutions that provide high-quality services in the Kingdom of Saudi Arabia [9]. Dental implant treatment is provided to patients by the faculty and consultants in the specialty clinic of the University Dental Hospital, as well as by residents of the postgraduate resident and fellowship programs. This study aimed to measure the alveolar bone thickness and crestal bone height in the esthetic zone of maxillary anterior teeth using CBCT to investigate its effect on surgical planning for dental implant placement in adult patients treated at King Abdulaziz University.

## Materials and methods

Study design and setting

Ethical approval was obtained from the Research Ethics Committee (REC), Faculty of Dentistry, King Abdulaziz University (# 288-09-21). A retrospective descriptive cross-sectional study was performed over four months to measure the alveolar bone thickness and crest bone height in the aesthetic zone of the maxillary anterior teeth. Recent CBCT scans from 870 adult male and female patients were obtained from the database at the Radiology Department, University Dental Hospital, King Abdulaziz University, Jeddah, KSA. CBCT examinations were taken previously for different clinical indications.

Sample size, inclusion criteria, and exclusion criteria

The sample size calculation was performed using Epi Info StatCal software version 7 (https://www.cdc.gov/epiinfo/user-guide/statcalc/statcalcintro.html). For the calculation, a 95% confidence level and an expected frequency of 10% were used to indicate a significant difference and effect within the representative population. A convenient sampling method was used. The inclusion criteria were subjects of both genders, aged between 20 and 70 years old, and the presence of all maxillary anterior teeth. The exclusion criteria included subjects who have implants or root canal treatment in the anterior teeth, missing one or more of the anterior teeth, individuals with a periodontally compromised dentition, CBCTs with poor quality, or CBCTs with voxels sizes more than 0.25 cubic millimeters (mm^3^). A total of 119 CBCT scans, from adults aged 18-65 years, with bilateral permanent maxillary anterior teeth present, were included in the study.

Measurements

Four independently trained and calibrated dentists performed the measurements on the CBCT scans. Measurement of the right upper canines (RUC), right upper lateral incisors (RUL), and right upper central incisors (RUCI) and left upper canines (LUC), left upper lateral incisors (LUL), and left upper central incisors (LUCI) were included in the analysis. Inter-examiner reproducibility was assessed using Kappa statistics. 

Radiographic assessment

**Figure 1:**
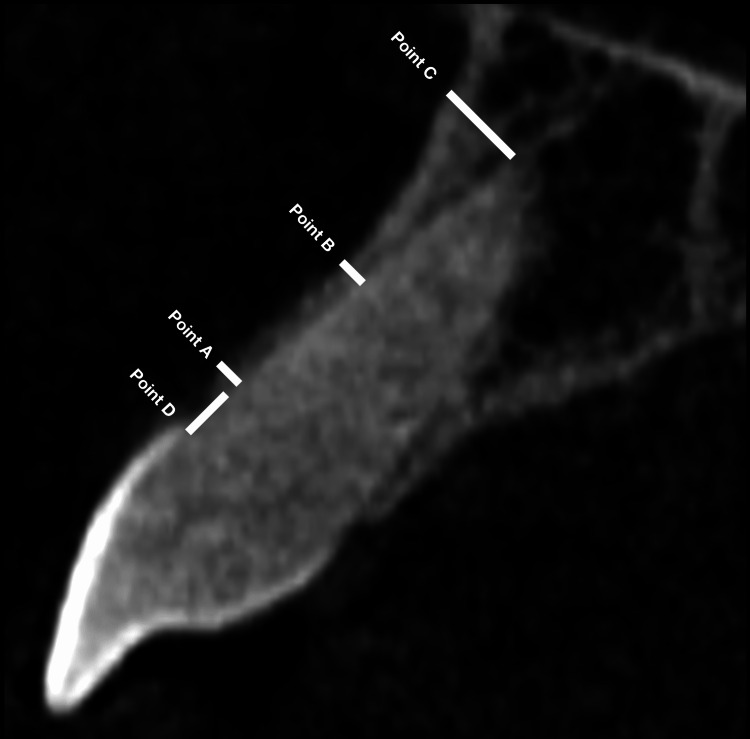
CBCT analysis of the bone thickness of the facial plate and the crestal bone height in a sagittal view from the facial aspect of the tooth root Measurements Point A: from the facial plate at the level of the bone crest to the coronal third of the root; Point B: from the facial plate at the level of the bone crest to the mid-root surface; Point C: from the facial plate at the level of the bone crest to the apical third of the root; Point D: from the cementoenamel junction (CEJ) to the alveolar crest CBCT: cone beam computed tomography

All CBCT images were obtained using an iCAT scanner (Imaging Sciences International, Hatfield, PA). All CBCT images were of mAs 5, kVp 120 and the voxel size of each image was 0.25 mm^3^ or less. For each CBCT image, a sagittal view of the root was used for measurement (Figure 1). The facial plate thickness of the alveolar bone was measured for each tooth. Reference points at three locations were measured for the alveolar bone facial thickness using a digital caliper. Point A was measured from the facial plate at the level of the bone crest to the coronal third of the root. Point B was measured from the facial plate at the level of the bone crest to the mid-root surface. Point C was measured from the facial plate at the level of the bone crest to the apical third of the root. All measurements were in mm. Measurements of crestal bone height loss (Point D) were done from the distance from the cementoenamel junction (CEJ) to the alveolar crest. The measurements were done using the same digital caliper on the same sagittal view for thickness measurements; all measurements were in mm. Descriptive and comparative analyses were performed for the quantitative data using SPSS software (IBM Corp., Armonk, NY). Independent-sample t-tests and correlation tests were used to analyze the collected data. The significance level was set at 95%. Statistical significance was set as P-value < 0.05.

## Results

Alveolar bone plate thickness

Average Thickness

Regarding the central incisors (CI), around 84% presented with an average alveolar bone facial thickness of <1.5 mm, 14% presented with facial bone thickness between 1.5 and 2 mm, and only 2% presented with a thickness of >2 mm. For the lateral incisors (LI), 90% presented with an average facial bone thickness of <1.5 mm, while 5% and 4% presented with an average thickness of 1.5-2 mm and >2 mm, respectively. Among the canines (CA), 100% had a facial bone thickness of <1.5 mm (Table 1).

**Table 1:** The percentage and frequency of the bone thickness of the facial plate within each category * <1.5: less than required thickness, 1.5–2: minimally required thickness, and >2: preferable required thickness CI = Central incisors, LI = Lateral incisors, CA = Canines

Categories of facial plate thickness (mm)*	CI n (%)	LI n (%)	CA n (%)
<1.5	84%	90%	100%
1.5-2	14%	5%	0%
>2	2%	4%	0%

**Figure 2:**
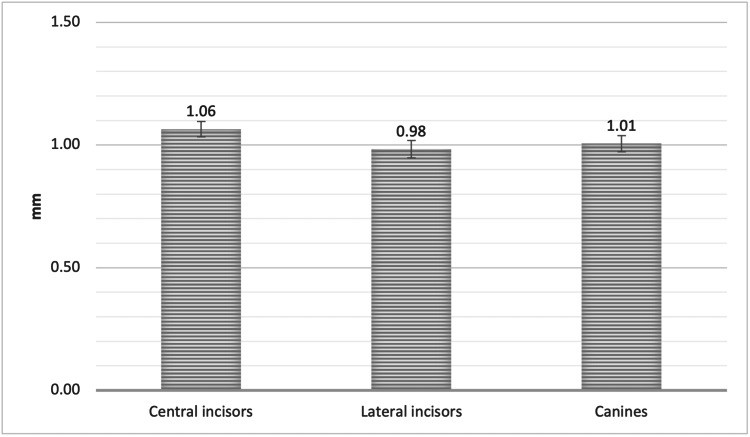
The mean bone thickness of the alveolar facial plate, measured from Points A, B, and C of the CI, LI, and CA * SEM = Standard error of the mean CI = Central incisors, LI = Lateral incisors, CA = Canines

The mean facial plate bone thickness, measured from the three horizontal points (average thickness at Point A, Point B, and Point C), in the sagittal view of the CBCT, was 1.06 ± 0.30 mm, 0.98 ± 0.34 mm, and 1.01 ± 0.31 mm for the upper central incisors (UCI), upper lateral incisors (ULI), and upper canines (UC), respectively, with no significant difference (Figure 2).

**Figure 3:**
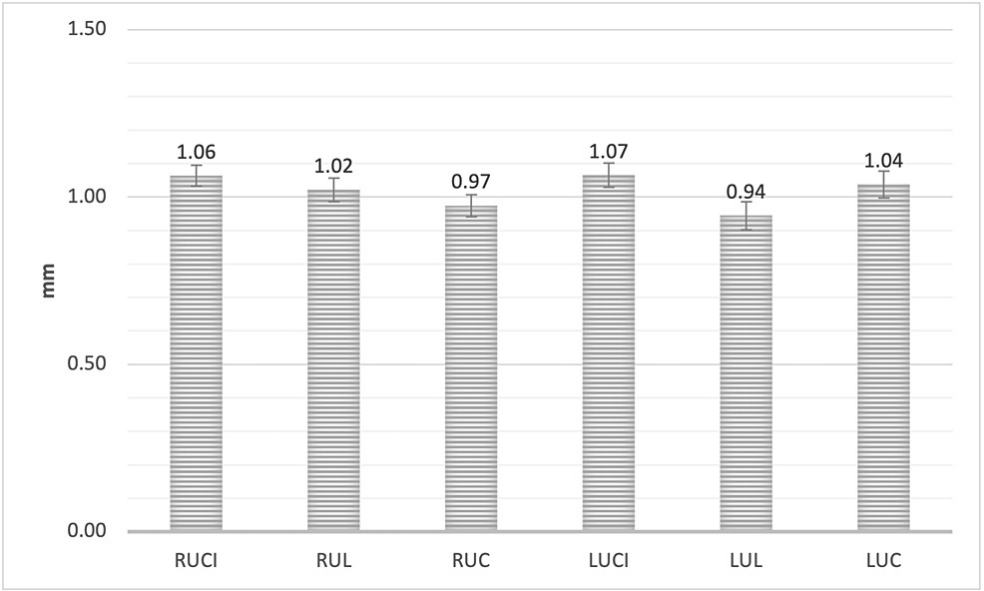
The mean bone thickness of the alveolar facial plate, measured from Points A, B, and C for all maxillary anterior teeth * SEM = Standard error of the mean RUCI = Right upper central incisors, RUL = Right upper lateral incisors, RUC = Right upper canines, LUCI = Left upper central incisors, LUL = Left upper lateral incisors, LUC = Left upper canines

Furthermore, the measurements were 1.06 ± 0.32 mm, 1.02 ± 0.36 mm, and 0.97 ± 0.34 mm for RUCI, RUL, and RUC, respectively. On the other hand, the LUCI, LUL, and LUC measured 1.07 ± 0.37 mm, 0.94 ± 0.42 mm, and 1.04 ± 0.41 mm, respectively. The mean alveolar bone thickness of the facial plate of the RUCI and LUCI was significantly greater than that of RUC and LUL (P < 0.05, Figure 3).

Point A

**Figure 4:**
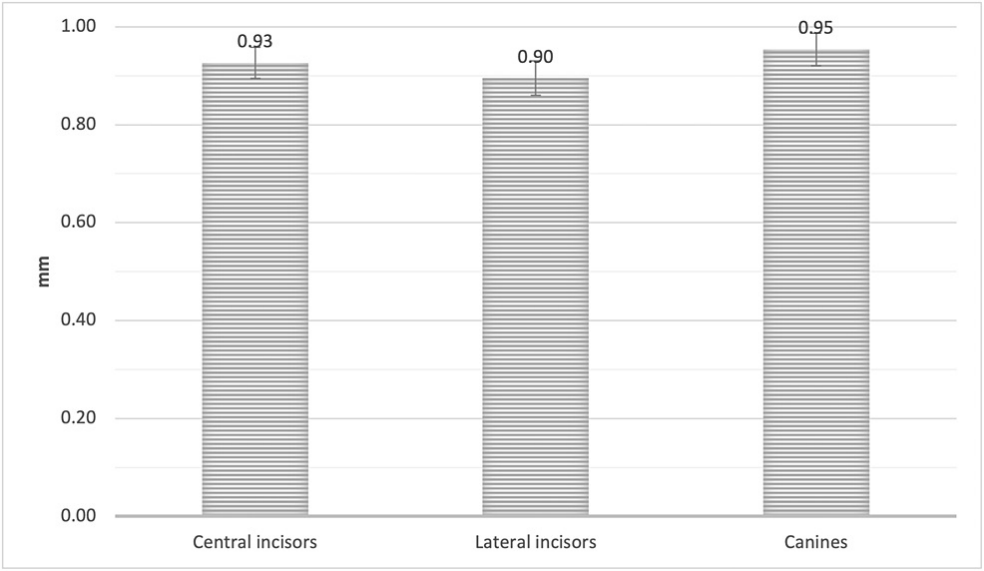
The mean bone thickness of the alveolar facial plate at point A, measured from the facial plate at the level of the bone crest to the coronal third of the root of the CI, LI, and CA CI = Central incisors, LI = Lateral incisors, CA = Canines

The mean facial plate bone thickness measured from the facial plate at the level of the bone crest to the coronal third of the root (Point A) in the sagittal view of the CBCT was 0.93 ± 0.24 mm, 0.90 ± 0.30 mm, and 0.95 ± 0.28 mm for UCI, ULI, and UC, respectively, with no significant difference (Figure 4).

**Figure 5:**
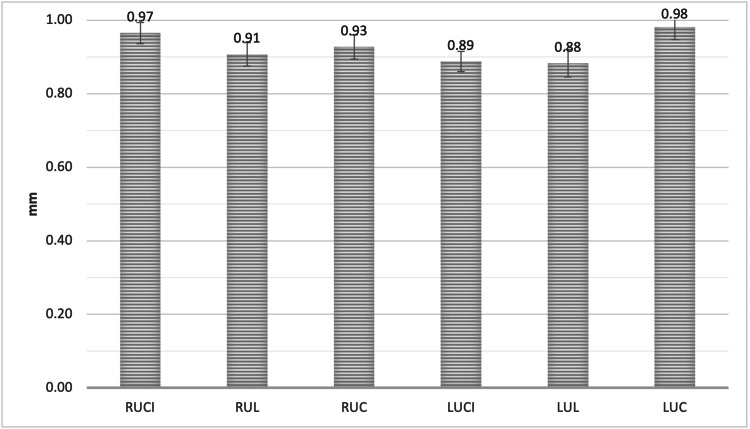
The mean bone thickness of the alveolar facial plate at point A, measured from the facial plate at the level of the bone crest to the coronal third of the root for all maxillary anterior teeth RUCI = Right upper central incisors, RUL = Right upper lateral incisors, RUC = Right upper canines, LUCI = Left upper central incisors, LUL = Left upper lateral incisors, LUC = Left upper canines

Furthermore, the point A measurement was 0.97 ± 0.30 mm, 0.91 ± 0.33 mm, and 0.93 ± 0.34 mm, for RUCI, RUL, and RUC, respectively. For LUCI, LUL and LUC, the measurement was 0.89 ± 0.29 mm, 0.88 ± 0.38 mm, and 0.98 ± 0.35mm, respectively. RUC showed a significantly greater mean alveolar facial plate bone thickness at point A when compared to LUCI (P < 0.05, Figure 5).

Point B

**Figure 6:**
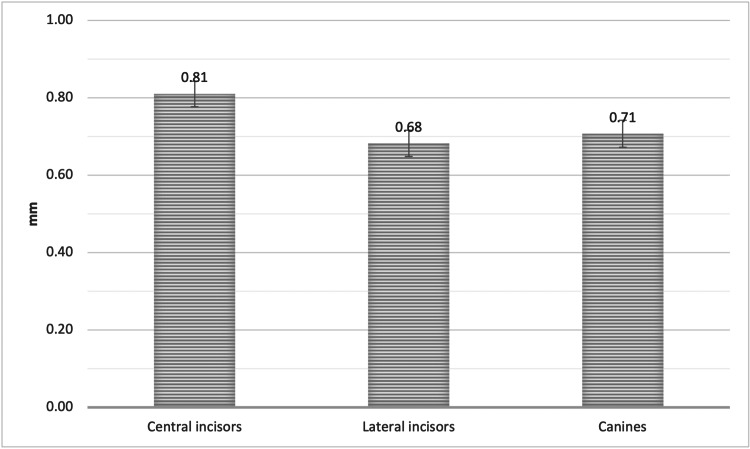
The mean bone thickness of the alveolar facial plate at point B, measured from the facial plate at the level of the bone crest to the mid-root surface of the CI, LI, and CA CI = Central incisors, LI = Lateral incisors, CA = Canines

The mean facial plate bone thickness, measured at Point B, to the level of the mid-root surface in the sagittal view of the CBCT was 0.81± 0.34 mm, 0.68 ± 0.35 mm, and 0.71 ± 0.36 mm for the UCI, ULI, and UC, respectively. The mean facial plate bone thickness of the UCI was significantly greater than that of the ULI and UC at Point B (P < 0.05, Figure 6).

**Figure 7:**
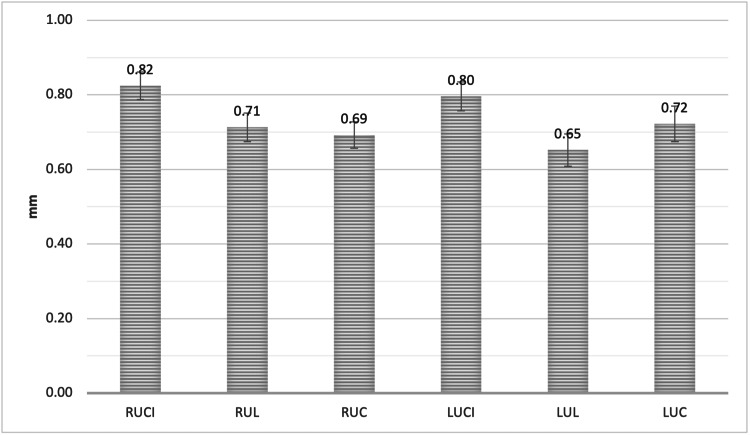
The mean bone thickness of the alveolar facial plate at point B, measured from the facial plate at the level of the bone crest to the mid-root surface for all maxillary anterior teeth RUCI = Right upper central incisors, RUL = Right upper lateral incisors, RUC = Right upper canines, LUCI = Left upper central incisors, LUL = Left upper lateral incisors, LUC = Left upper canines

Furthermore, the Point B measurement was 0.82 ± 0.38 mm, 0.71 ± 0.40 mm, and 0.69 ± 0.37 mm for RUCI, RUL, and RUC, respectively. For the LUCI, LUL, and LUC, the measurement was 0.80 ± 0.40 mm, 0.65 ± 0.45 mm, and 0.72 ± 0.49 mm, respectively. The RUCI showed significantly greater mean alveolar facial plate bone thickness at Point B when compared to RUL, RUC, and LUL (P < 0.05). Furthermore, LUCI showed significantly greater mean alveolar facial plate bone thickness at Point B when compared to RUC and LUL (P < 0.05, Figure 7).

Point C

**Figure 8:**
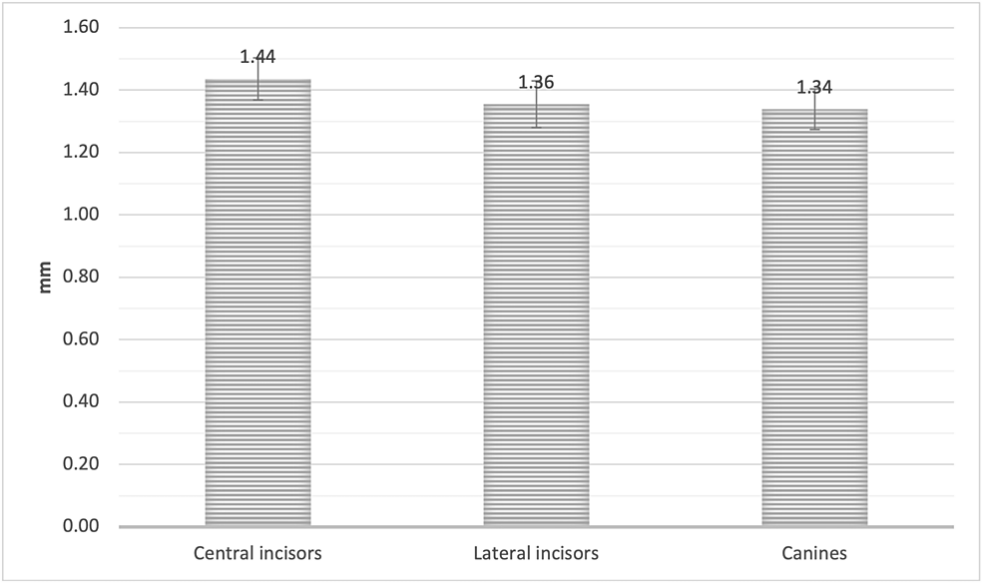
The mean bone thickness of the alveolar facial plate at point C, measured from the facial plate at the level of the bone crest to the apical third of the root for CI, LI, and CA CI = Central incisors, LI = Lateral incisors, CA = Canines

The mean facial plate bone thickness was measured at the apical third of the root (Point C) in the sagittal view of the CBCT. The measurement was 1.44 ± 0.70 mm, 1.36 ± 0.77 mm, and 1.34 ± 0.67 mm, for UCI, ULI, and UC, respectively, with no significant difference (Figure 8).

**Figure 9:**
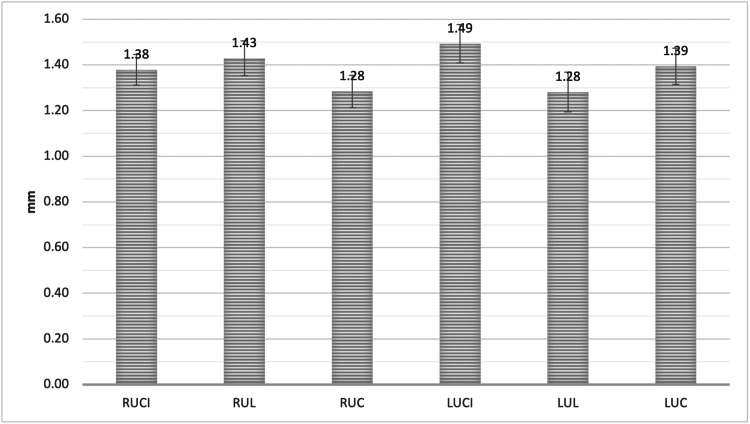
The mean bone thickness of the alveolar facial plate at point C, measured from the facial plate at the level of the bone crest to the apical third of the root for all maxillary anterior teeth RUCI = Right upper central incisors, RUL = Right upper lateral incisors, RUC = Right upper canines, LUCI = Left upper central incisors, LUL = Left upper lateral incisors, LUC = Left upper canines

The mean Point C measurement was 1.38 ±0.69 mm, 1.43 ± 0.79 mm, and 1.28 ± 0.73 mm, for RUCI, RUL, and RUC, respectively. For LUCI, LUL, and LUC, the measurement was 1.49 ± 0.87 mm, 1.28 ± 0.90 mm, and 1.39 ± 0.82 mm, respectively, with no significant difference (Figure 9).

Table 2 shows the mean facial plate bone thickness of the upper anterior teeth.

**Table 2:** The facial plate mean thickness of the upper anterior teeth (N = 119) * P-value < 0.05 CI = Central incisors, LI = Lateral incisors, CA = Canines

Distance (mm)	CI Mean± Std.	P-value*	LI Mean ± Std.	P-value*	CA Mean ± Std.	P-value*
Point A	0.93 ± 0.24		0.9 ± 0.30		0.95 ± 0.28	
Point B	0.81 ± 0.34	<0.05	0.68 ± 0.35	<0.05	0.71 ± 0.36	<0.5
Point C	1.44 ± 0.70		1.36 ± 0.77		1.34 ± 0.67	
Average Thickness	1.06 ± 0.30		0.98 ± 0.34		1.01 ± 0.31	

Table 3 shows the mean facial plate bone thickness difference for the upper anterior teeth on the right versus the left side.

**Table 3:** The mean differences in the bone thickness of the facial plate of the upper anterior teeth on the right and left sides (N = 119) For all comparisons, P > 0.05. CI = Central incisors, LI = Lateral incisors, CA = Canines

Distance (mm)	CI (N = 238)		LI (N = 238)		CA (N = 238)	
	Right	Left	Right	Left	Right	Left
Point A	0.97 ± 0.30	0.89 ± 0.29	0.91 ± 0.33	0.88 ± 0.38	0.93 ± 0.34	0.98 ± 0.35
Point B	0.82 ± 0.38	0.80 ± 0.40	0.71 ± 0.40	0.65 ± 0.45	0.69 ± 0.37	0.72 ± 0.49
Point C	1.38 ± 0.69	1.49 ± 0.87	1.43 ± 0.79	1.28 ± 0.90	1.28 ± 0.73	1.39 ± 0.82
Average Thickness	1.06 ± 0.32	1.07 ± 0.37	1.02 ± 0.36	0.94 ± 0.42	0.97 ± 0.34	1.04 ± 0.41

Alveolar Bone Height Loss - Point D

The mean reduction in alveolar bone height was measured as the distance from the cementoenamel junction (CEJ) to the alveolar crest at the apical third of the root (Point D) in the sagittal view of the CBCT. Regarding the central incisors (CI), around 62% presented with an alveolar bone height distance of <3 mm and 38% presented with an alveolar bone height distance of >3 mm. For the lateral incisors (LI), 61% presented with an alveolar bone height distance of <3 mm while 39% presented with an alveolar bone height distance of >3. Among the canines (CA), 52% had an alveolar bone height distance of <3 mm and 48% presented with an alveolar bone height distance of > 3 mm (Table 4).

**Table 4:** The percentage and frequency of the loss of the alveolar bone height within each category * <3: preferable required height, >3: less than the required height CI = Central incisors, LI = Lateral incisors, CA = Canines

Categories of alveolar bone height loss (mm)*	CI n (%)	LI n (%)	CA n (%)
<3	62%	61%	52%
>3	38%	39%	48%

**Figure 10:**
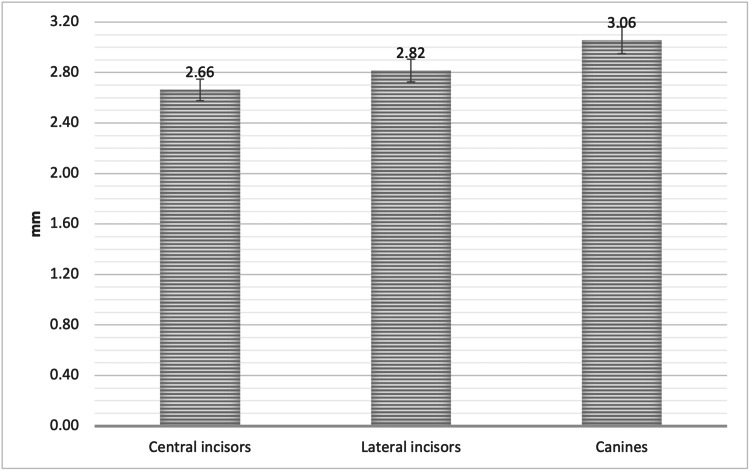
The mean alveolar bone height loss (Point D) as measured from the alveolar crest to the cementoenamel junction (CEJ) in the CI, LI, and CA CI = Central incisors, LI = Lateral incisors, CA = Canines

**Figure 11:**
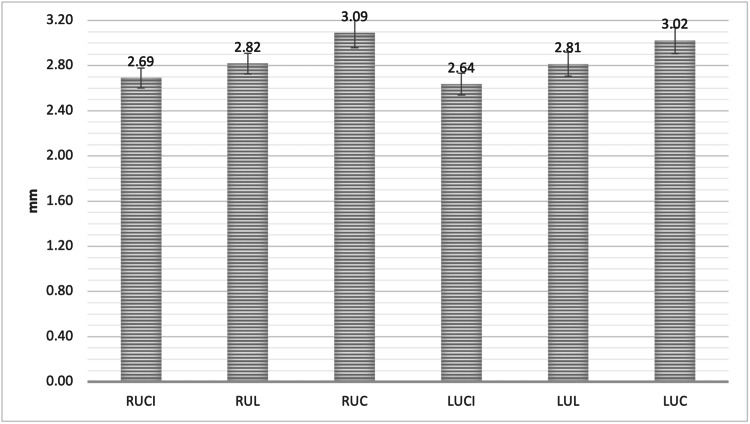
The mean alveolar bone height loss (Point D) as measured from the alveolar crest to the cementoenamel junction (CEJ) in all maxillary anterior teeth RUCI = Right upper central incisors, RUL = Right upper lateral incisors, RUC = Right upper canines, LUCI = Left upper central incisors, LUL = Left upper lateral incisors, LUC = Left upper canines

For the UCI, ULI, and UC, the measurement was 2.66 ± 0.88 mm, 2.82 ± 0.92 mm, and 3.06 ± 1.09 mm, respectively. The UC demonstrated a significantly greater mean reduction in alveolar bone height when compared to the UCI at point D. The point D measurement was 2.69 ± 0.91 mm, 2.82 ± 0.94 mm, and 3.09 ± 1.35 mm, for RUCI, RUL, and RUC, respectively. For the LUCI, LUL, and LUC, the measurement was 2.64 ± 0.99 mm, 2.81 ± 1.11 mm, and 3.02 ± 1.19 mm, respectively. The RUC and LUC showed a significantly greater reduction in the mean alveolar bone height when compared to RUCI and LUCI at point D (P < 0.05, Figure 10, Figure 11, and Table 5).

**Table 5:** The mean differences in the alveolar bone height reduction of the upper anterior teeth and on the right and left sides (N = 119) For all comparisons, P > 0.05 *P-value < 0.05 CI = Central incisors, LI = Lateral incisors, CA = Canines

Distance (mm)	*CI (N= 238)		LI (N= 238)		*CA (N=238)	
Average	2.66 ± 0.88		<0.05		2.82 ± 0.92	
Side	Right	Left	Right	Left	Right	Left
Average	2.69 ± 0.91	2.64 ± 0.99	2.82 ± 0.94	2.81 ± 1.11	3.09 ± 1.35	3.02 ± 1.19

Alveolar Bone Plate Thickness and Alveolar Bone Height Loss

**Figure 12:**
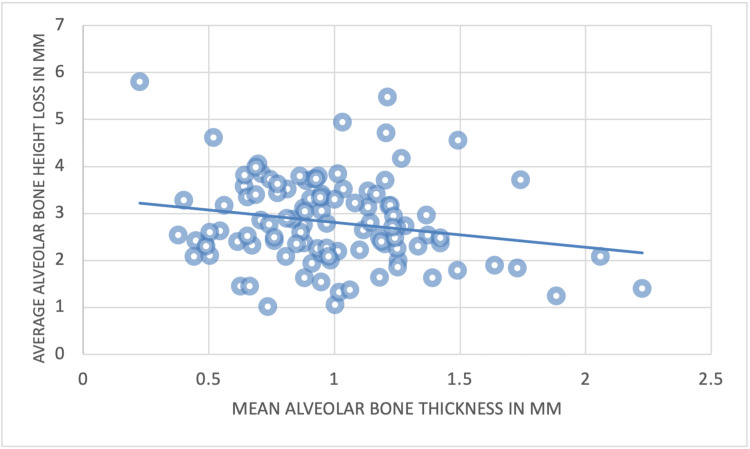
The correlation between the mean alveolar bone plate thickness and the loss of alveolar bone height

The Pearson correlation coefficient analysis was performed to demonstrate the correlation between the alveolar bone plate thickness and the loss of alveolar bone height. There was no significant correlation between the alveolar bone plate thickness and the loss of alveolar bone height on the CI and CA. In contrast, a significant negative correlation was noted between the alveolar bone plate thickness and the loss of alveolar bone height at the LI (Figure 12).

Gender

Of the 119 CBCT images, 42 images were from male patients and 77 were from female patients. A comparison was performed between both groups and the results indicated that there was no significant difference in the average alveolar plate bone thickness overall or at each of the three reference points. Furthermore, there was no significant difference in the reduction in alveolar bone height between men and women (Table 6).

**Table 6:** Comparison of the bone thickness of the facial plate of the upper anterior teeth according to gender (N = 119) * Measures were in millimeters. ** Independent t-test CI = Central incisors, LI = Lateral incisors, CA = Canines

Tooth	Point measured*	Gender (Mean ± SD)		P-value**
		Female	Male	
CI	Point A	0.92 ± 0.24	0.93 ± 0.23	0.80
	Point B	0.80 ± 0.32	0.81 ± 0.37	0.89
	Point C	1.39 ± 0.64	1.5 ± 0.77	0.39
	Average thickness	1.04 ± 0.30	1.088 ± 0.34	0.49
	Point D	1.1 ± 0.41	1.16 ± 0.45	0.45
LI	Point A	0.86 ± 0.26	0.94 ± 0.35	0.20
	Point B	0.67 ± 0.33	0.69 ± 0.37	0.87
	Point C	1.37 ± 0.83	1.33 ± 0.65	0.81
	Average thickness	0.96 ± 0.34	1.02 ± 0.36	0.42
	Point D	1.02 ± 0.47	1.01 ± 0.41	0.88
CA	Point A	0.95 ± 0.25	0.95 ± 0.32	0.92
	Point B	0.73 ± 0.36	0.67 ± 0.34	0.32
	Point C	1.29 ± 0.74	1.42 ± 0.48	0.34
	Average thickness	0.98 ± 0.30	1.05 ± 0.31	0.29
	Point D	1.01 ± 0.47	1.04 ± 0.33	0.73

Age

Table 7 summarizes the results of the Pearson correlation coefficient analysis.

**Table 7:** Correlation coefficients between age and facial plate thickness and the loss of the alveolar bone height, measured from the three reference points of the maxillary anterior teeth * Pearson correlation is significant at the 0.05 level. CI = Central incisors, LI = Lateral incisors, CA = Canines

Teeth	Points Measured	Age	
		r	P-Value
CI	Point A*	0.2	0.03
	Point B	0.04	0.6
	Point C*	-0.22	0.02
	Average Thickness	-0.13	0.16
	Point D*	0.32	0.0005
LI	Point A	0.17	0.07
	Point B	0.17	0.06
	Point C	-0.16	0.08
	Average Thickness	-0.13	0.16
	Point D*	0.26	0.005
CA	Point A	0.18	0.051
	Point B	-0.07	0.43
	Point C*	-0.2	0.009
	Average Thickness	-0.16	0.08
	Point D	0.09	0.33

**Figure 13:**
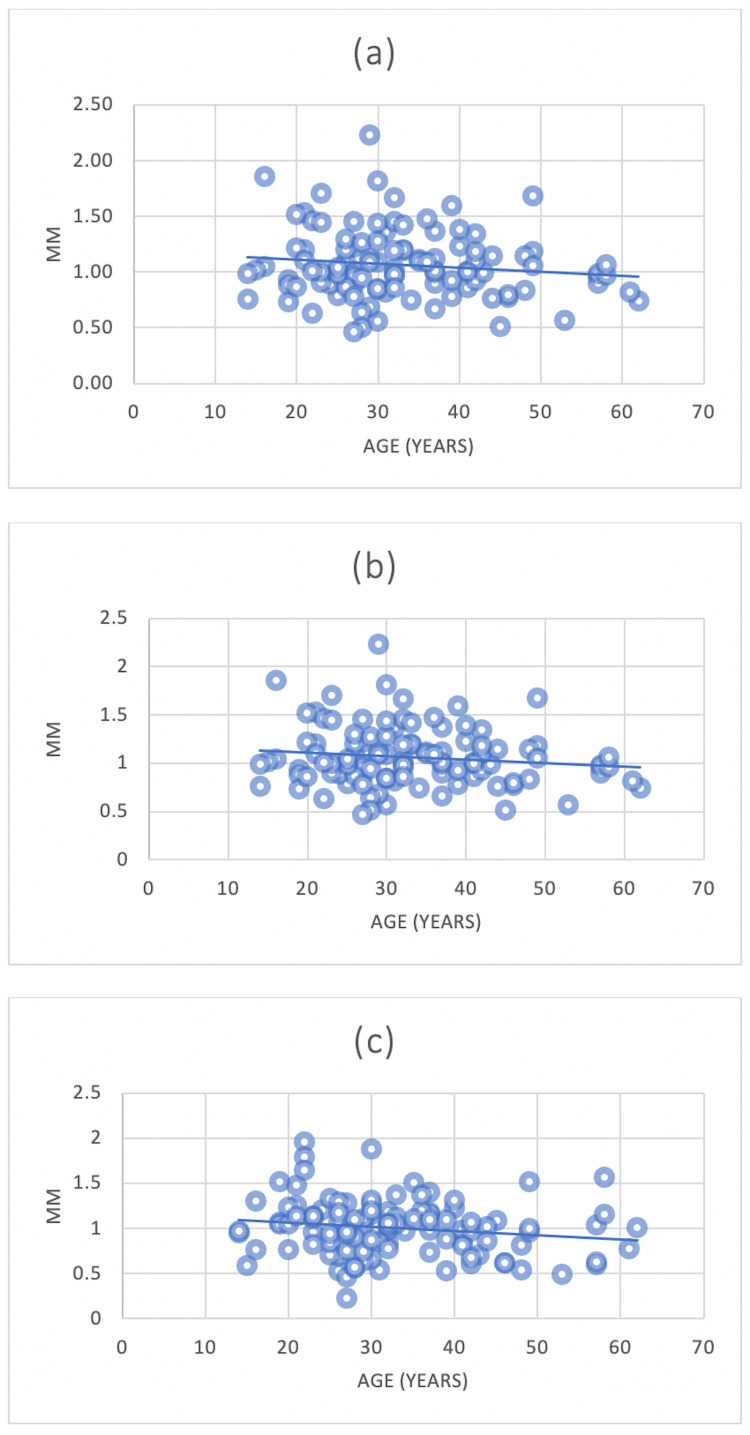
The correlation between the mean alveolar bone plate thickness of CI, LI, and CA with age (a) = Central incisors (CI) and age; (b) = Lateral incisors (LI) and age; (c)= Canines (CA) and age

The results demonstrated the correlation between age and the alveolar bone plate thickness and the loss of alveolar bone height. There was no significant correlation between the mean alveolar palate thickness of the anterior teeth and age (Figures 13a-13c).

**Figure 14:**
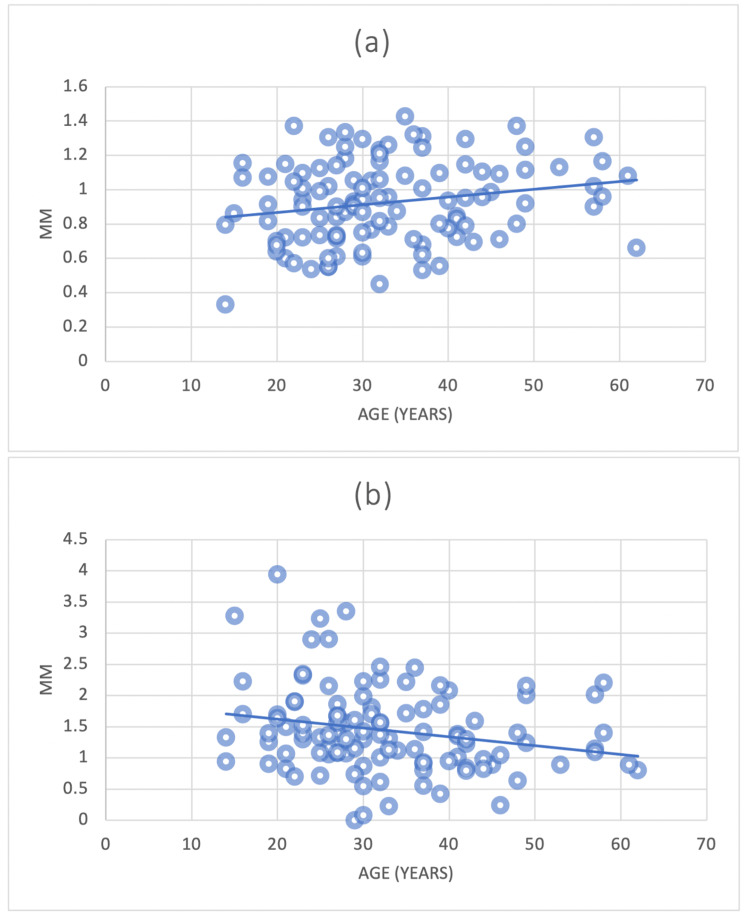
The correlation between the mean alveolar bone plate thickness of CI (point A and point C) and age (a) = Central incisors (CI) Point A and age; (b)= Central incisors (CI) Point C and age

**Figure 15:**
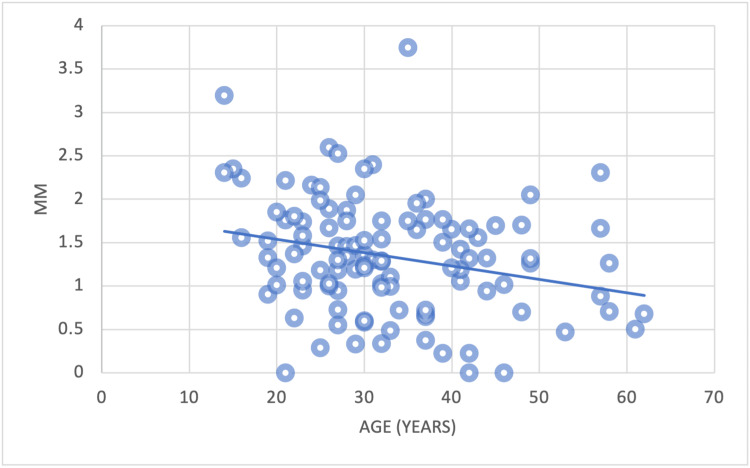
The correlation between the mean alveolar bone plate thickness of CA (point C) and age CA = Canines

In contrast, a significant correlation was noted between the alveolar plate thickness reduction and increasing age at the CI, measured at Point A and Point C. The same correlation at Point C was also detected for CA (Figures 14a-14b, 15).

**Figure 16:**
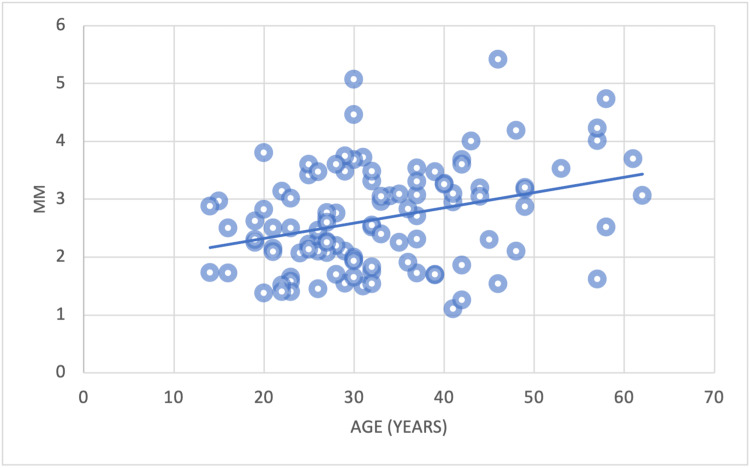
The correlation between the mean alveolar bone plate height reduction of CI and age CI = Central incisors

**Figure 17:**
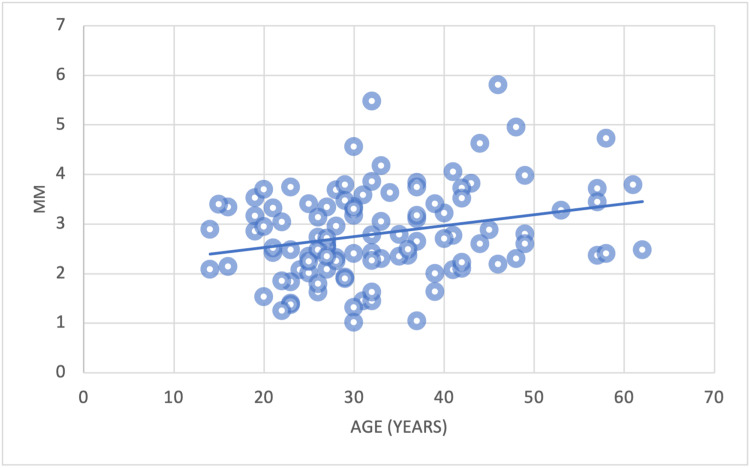
The correlation between the mean alveolar bone plate height reduction of LI and age LI = Lateral incisors

There was a significant positive correlation between the reduction in alveolar bone height (Point D) and age observed at CI and LI (Figures 16, 17). Conversely, there was no correlation among CA.

## Discussion

Our study aimed to investigate the alveolar bone thickness and crestal bone height in the esthetic zone of maxillary anterior teeth using CBCT and showed that most of the anterior teeth exhibited less than 1.5 mm facial bone plate thickness. Of note, our data excluded patients who have dental implants or root canal treatment in the anterior teeth, those who have one or more missing anterior teeth, and those who are periodontally compromised. Our findings concurred with the results of Sheerah et al.’s 2019 study [10].

Patients who come with “hopeless” teeth due to periodontal or restorative reasons are expected to have an even greater reduction in the thickness of their facial plate and crestal bone height due to local factors. Moreover, vertical and horizontal bone loss is expected after teeth extraction [7]. This is very important, as dental implant placement following teeth extraction will rely on the existence of a buccal bone with enough thickness and height [4]. When dental implants are used to treat missing maxillary anterior teeth, the height and thickness of the alveolar bone plate and the soft tissue biotype should be considered [11]. The mean thickness of the alveolar bone plate was significantly greater in RUCI and LUCI when compared to RUC and LUL. These results are in line with the findings of Soumya et al. [12]. and Sheerah et al. [10].

The mean alveolar bone plate thickness at Point B was significantly higher in CI when compared to LI and CA. This may increase the risk of dehiscence and fenestration at the mid-root level of the LI and CA and should be assessed when considering dental implants. This may be due to the wider root diameter, the buccal prominence of the CA, and the concavity of the maxilla in the LI regions.

When measuring the mean thickness of the facial plate at Point C, there was no significant difference between any of the anterior teeth. The reason may be due to the normal anatomy of the region, as the base of the maxilla is wider than the coronal third of the root. The mean reduction in the alveolar bone plate height at Point D was significantly greater in the CA than in the CI. As such, when providing dental implants in the CA regions, guided bone regeneration or utilizing pink porcelain to simulate the gingiva may need to be considered.

The alveolar bone plate thickness did not differ between men and women. This finding contrasted with the results reported by Sheerah et al. [10].

In their 2019 study, they reported that males have a thicker alveolar plate than females. Our findings on the loss of alveolar height exhibited similar results to Sheerah et al.’s 2019 study, in which no significant difference in the reduction of the alveolar crestal bone height was detected between males and females [10].

The age correlation was positively established at the CI for Points A, C, and D, the LI at Point D, and the CA at Point C. This may contribute to a higher frequency of fenestrations and dehiscence in these sites with age, and thus affect the outcomes of dental implants among older patients. Furthermore, this increased risk of bony defects may affect the treatment plan. Guided bone regeneration and “pink restorative solutions” may need to be considered.

Alveolar bone thickness in maxillary anterior teeth has exhibited a thin pattern [13]. Crestal bone height, proximal contact length, and tooth shape were associated with the appearance of gingival papilla [14]. Papilla was present in almost 100% of the cases that demonstrated 5 mm or less measurement from the contact point to the crest of the bone [15].

CBCT was useful for periodontal bone loss detection and the visualization of defects was improved [16]. CBCT assisted in the planning of implant cases and reduced the deviation of surgical implant placement [17] The morphology of alveolar bone was different from healthy and periodontitis patients when measured and analyzed by CBCT [18].

Dental implant placement depends on the morphology of the bone. In the Chinese population, CBCT analysts showed 26.07% of the sites with fenestrations [19]. It is consistent with our study results, as fenestrations tend to be present and increase with age.

The dehiscence rate reached 51.6% for anterior teeth in Asians. However, there was no significant difference between alveolar facial plate thickness between the right and left sides. Furthermore, the distance between CEJ and bone crest increased with age, and rare sites were found with 2 mm facial plate width [20]. These data were smiler to our study except they showed a higher dehiscence rate than our study and other studies.

The inter-dental implant distance is important to respect to avoid bone loss [21]. In a virtual implant placement study, CBCT was used to simulate dental implant placement in the cingulum position for ideal restoration. It was found that around 20% of the cases ended up with fenestrations [22]. These data support our study as guided bone regeneration and “pink restorative solutions” may need to be considered. An experimental study on a dog showed buccal and lingual bone resorption after teeth extraction [23]. Clinically, bone resorption is expected after teeth extraction [24]. A thin facial plate left following teeth extraction supports the need for CBCT analysis to determine the need for grafting.

Few maxillary teeth had greater alveolar facial plate thickness than 1 mm. The bone tends to get thicker in a coronal apical direction and in an anterior-posterior direction [25]. Teeth position within the alveolar ridge didn't have a significant impact on the facial plate thickness [26]. Our study didn't analyze teeth inclination or crowding. It is recommended to include a larger sample of teeth and includes these criteria to allow a further understanding of alveolar plate thickness.

Dental implant treatment is dependent on the presence of the facial plate. In one in every four cases, flapless implant placement is not feasible due to the thin facial plate [27]. Facial plate width variation from tooth to tooth has been documented. Furthermore, it is also variable at the same tooth level, and it featured heterogeneity [28]. There has been a positive correlation between the facial and palatal plate of the bone and the overlying soft tissue thickness. Furthermore, the labial plate bone showed a positive correlation with the labiopalatal width of the alveolar socket [29]. Gingival phenotype showed a significant correlation with gingival recession in the anterior area [30]. Therefore, it is another reason for the importance of the presence of a thick facial plate prior to dental implant placement.

The study has several limitations. Our data did not exclude CBCT images of medically compromised patients and didn't include any analysis of teeth position within the arch. However, because of that, our data is a true representation of the population presented to the facility. Therefore, our analysis contributes specific findings that may improve treatment planning and management of the targeted population at King Abdulaziz University. The correlation between alveolar bone plate thickness and alveolar bone height loss was addressed as a new parameter in this study and the literature has limited studies measuring that correlation. Lateral incisors showed a significant negative correlation between alveolar bone thickness and the loss of alveolar bone height. This correlation is another contributing factor to the need for careful assessment when replacing lateral incisors with dental implants. The main purpose of this study was to measure the crestal bone height loss and alveolar bone thickness in the esthetic area to determine the factors that may influence surgical planning for dental implant placement. It is recommended to include a larger population and more variables such as an analysis of teeth positions in the dental arch. It is also recommended to include the ethnicity of the participants to investigate the impact of their background on the morphology of the bone. Patients with periodontitis should also be included and compared to healthy patients. Teeth length and width should be documented in future studies to establish a correlation between tooth size and alveolar bone. This will also aid in periodontal disease diagnosis by measuring the percentage of bone loss in relation to teeth length to determine the stage of classification. The study was the first study of its kind in Jeddah, Saudi Arabia. It has included narrower exclusive criteria than previous studies. The results of this study may potentially provide significant relevance to further cases related to dental implant surgery. This study is a starting point for future research in dental implant treatment and management in the esthetic zone in Saudi Arabia.

## Conclusions

Alveolar bone plate thickness and the reduction in crestal bone height in maxillary anterior teeth exhibited significant differences. Additional care and detailed assessment before dental implant placement should be considered when replacing missing LI and CA. LI showed a negative correlation between alveolar bone thickness and the loss of alveolar bone height. The frequency of fenestrations and dehiscence increases with age. Guided bone regeneration or “pink restorative solutions” may need to be considered as part of treatment planning. The examination of bone morphology with CBCT is critical for accurate diagnosis and management of "hopeless" maxillary anterior teeth with dental implants.
